# Semi-Quantitative Parameter Analysis of DCE-MRI Revisited: Monte-Carlo Simulation, Clinical Comparisons, and Clinical Validation of Measurement Errors in Patients with Type 2 Neurofibromatosis

**DOI:** 10.1371/journal.pone.0090300

**Published:** 2014-03-04

**Authors:** Alan Jackson, Ka-Loh Li, Xiaoping Zhu

**Affiliations:** Wolfson Molecular Imaging Centre, The University of Manchester, Manchester, United Kingdom; University of Kentucky, United States of America

## Abstract

**Purpose:**

To compare semi-quantitative (SQ) and pharmacokinetic (PK) parameters for analysis of dynamic contrast enhanced MR data (DCE-MRI) and investigate error-propagation in SQ parameters.

**Methods:**

Clinical data was collected from five patients with type 2-neurofibromatosis (NF2) receiving anti-angiogenic therapy for rapidly growing vestibular schwannoma (VS). There were 7 VS and 5 meningiomas. Patients were scanned prior to therapy and at days 3 and 90 of treatment. Data was collected using a dual injection technique to permit direct comparison of SQ and PK parameters. Monte Carlo modeling was performed to assess potential measurement errors in SQ parameters in persistent, washout, and weakly enhancing tissues. The simulation predictions for five semi-quantitative parameters were tested using the clinical DCE-MRI data.

**Results:**

In VS, SQ parameters and *K^trans^* showed close correlation and demonstrated similar therapy induced reductions. In meningioma, only the denoised Signal Enhancement Ratio (R_se1/se2(DN)_) showed a significant therapy induced reduction (p<0.05). Simulation demonstrated: 1) Precision of SQ metrics normalized to the pre-contrast-baseline values (MSE_rel_ and ∑MSE_rel_) is improved by use of an averaged value from multiple baseline scans; 2) signal enhancement ratio R_mse1/mse2_ shows considerable susceptibility to noise; 3) removal of outlier values to produce a new parameter, R_mse1/mse2(DN)_, improves precision and sensitivity to therapy induced changes. Direct comparison of in-vivo analysis with Monte Carlo simulation supported the simulation predicted error distributions of semi-quantitative metrics.

**Conclusion:**

PK and SQ parameters showed similar sensitivity to anti-angiogenic therapy induced changes in VS. Modeling studies confirmed the benefits of averaging baseline signal from multiple images for normalized SQ metrics and demonstrated poor noise tolerance in the widely used signal enhancement ratio, which is corrected by removal of outlier values.

## Introduction

Analysis of dynamic contrast enhanced MRI (DCE-MRI) data is commonly performed by applying pharmacokinetic (PK) models to changes in contrast agent concentration derived from observed changes in signal intensity (SI). A simpler approach is to perform direct analysis of changes in SI using one or more of a range of established semi-quantitative (SQ) descriptors.

Almost all early DCE-MRI studies employed simple SQ metrics derived by mathematical analysis of observed SI-time course data (SI-TC). With the growth of PK approaches the field has become dichotomized with consensus groups recommending PK analysis [1], [2], [3], [4] whilst, at the same time clinical radiologists are far more likely to use SQ metrics which have become essential clinical tools across a range of oncological applications [5], [6], [7], [8], [9], [10], [11], [12], [13], [14], [15], [16], [17], [18], [19], [20], [21], [22], [23], [24], [25], [26], [27]. This widespread adoption of SQ metrics reflects the simplicity of the approach, the ability to use straightforward, often slow, dynamic acquisition sequences with good spatial resolution and coverage, the wide availability of clinical analysis software and, most importantly, clear clinical evidence that the techniques are beneficial.

Despite widespread clinical adoption SQ parameters are commonly avoided for clinical trial applications in the belief that they are less biologically specific and more prone to variability than parameters derived from PK modeling, [2], [4] although, no detailed study of the behavior of SQ parameters or direct comparison of SQ and PK parameters has been presented. Many studies have however shown significant problems associated with PK derived parameters arising from the need for high temporal resolution sampling, accurate arterial input function definition and problems associated with curve fitting based analysis approaches which are limiting widespread implementation of DCE-MRI, particularly into multi-center studies [4], [28], [29], [30].

The signal enhancement ratio (R_mse1/mse2_) is particularly attractive SQ metric which is little affected by variation in tissue *T*_10_ values and has been shown to correlate closely with the redistribution rate constant (*k*_ep_), a commonly analyzed PK parameter [31]. This behavior identifies R_mse1/mse2_ as a potential simple and attractive surrogate of PK analyses, which has led to widespread adoption, particularly in breast cancer studies [9], [10], [11], [12], [13], [32], [33], [34], [35], [36].

Anxieties around the use of SQ parameters for quantitative studies have little or no substantive evidence base [37]. Multicenter clinical applications are common and the development of “normalized” SQ metrics represents an attempt to minimize variation across multi-center/multivendor acquisitions. Wider use of SQ parameters for clinical trials and multicenter studies would simplify data analysis and potentially improve implementation of DCE-MRI. However, before this could occur it is essential that we have a greater understanding of their ability to identify therapy induced changes, the comparative performance of SQ and PK derived biomarkers and their variability in the setting of multicenter studies.

Direct comparison of SQ and PK parameters is complicated by the differing acquisition approaches employed. SQ parameters are typically used in low temporal resolution data allowing the use of high spatial resolution and large volume coverage. In contrast, PK parameters depend on high temporal resolution to allow accurate fitting of the analytical model, which produces compromises in both spatial resolution and coverage. We have recently described a dual injection technique, ICR-DICE (**I**mproved **C**ontrast and spatial **R**esolution using **D**ual **I**njection **C**onrast **E**nhanced MRI) [38]. This uses two separate contrast enhanced dynamic acquisitions to provide a high temporal resolution dataset, ideal for identification of an arterial input function for PK mapping, and a high spatial resolution dataset for measurement of tissue residue functions allowing direct, pixel by pixel comparison of SQ and PK metrics in the same dataset.

This study uses a combination of Monte Carlo modeling and clinical ICR-DICE data from patients with type 2 Neurofibromatosis and was designed to address five basic issues:

1. What is the predicted effect of variations in signal intensity curve shape and signal to noise ratio on errors in the estimates of SQ parameters?
2. Do observations from the clinical DCE-MRI data support Monte-Carlo predictions?
3. Does the behavior of the signal enhancement ratio (R_mse1/mse2_) provide a satisfactory surrogate metric for formal PK based analyses?
4. What is the optimal number of samples to define pre-enhancement signal intensity on estimates of SQ parameters?
5. How do SQ and PK derived metrics compare in the detection of anti-angiogenic therapy induced changes in a single centre setting?

## Methods

### Choice of Semi-Quantitative Parameters

Candidate SQ parameters were categorized into 5 groups, each characterized by a similar mechanism of error propagation during parameter calculation. An additional novel parameter, designed to improve computational stability, was subsequently added. The final 6 SQ parameters were:

1. *Absolute MRI signal enhancement* (MSE) [39], (e.g., MSE_1min_ = SI_1min,post_−SI_pre_), where SI_1min,post_ is signal intensity at one minute post contrast agent (CA) injection and SI_pre_ is SI pre-injection. One commonly used example of this category is the maximum intensity change per time interval ratio (MITR) [40].
2. *Relative signal enhancement* (MSE_rel_) [14], [22], [41] which uses a baseline value for normalization in order to reduce the dependence on biological and imaging system variables, e.g., MSE_rel, 1min_ = (SI_1min post_−SI_pre_)/SI_pre_, where MSE_rel, 1min_ is MSE_rel_ at one minute post CA injection.
3. *The sum of MSE over a fixed post-injection duration*, (∑MSE). which can also be defined as the area under the enhancement curve (AUC) [1].
4. *The sum of MSE_rel_ over a fixed post-injection duration*, (∑MSE_rel_), which can also be defined as baseline-normalized AUC [1].
5. *Signal enhancement ratio*, (R_mse1/mse2_) commonly defined as the ratio of early to late contrast enhancement ratio [32], e.g., R_mse1/mse2_ = (SI_1min post_−SI_pre_)/(SI_5min post_−SI_pre_); Alternatively, defined as the ratio of late to early contrast enhancement, e.g., R_mse2/mse1_ = (SI_5min post_−SI_pre_)/(SI_1min post_−SI_pre_), which was also coined the name of SER (Signal Enhancement Ratio).
6. *Denoised Signal enhancement ratio* (R_mse1/mse2(DN_) Comparison of values in mse1, mse2, and R_mse1/mse2_ maps demonstrated that 3–4% of voxels have spurious values resulting from noise-induced near-zero values in mse2 maps whereas mse1≠0. To remove these outliers, thresholds of 0<R_mse1/mse2_<1.45 were used to exclude voxels outside the 95th percentile and R_mse1/mse2_ calculated as described above. Note that a median filter could be used to filter outliers, but may also affect high frequency components of the SER maps [42].

### Monte-Carlo Modeling of Measurement Errors

#### Generation of typical signal intensity-time course curves

**Figure 1:**
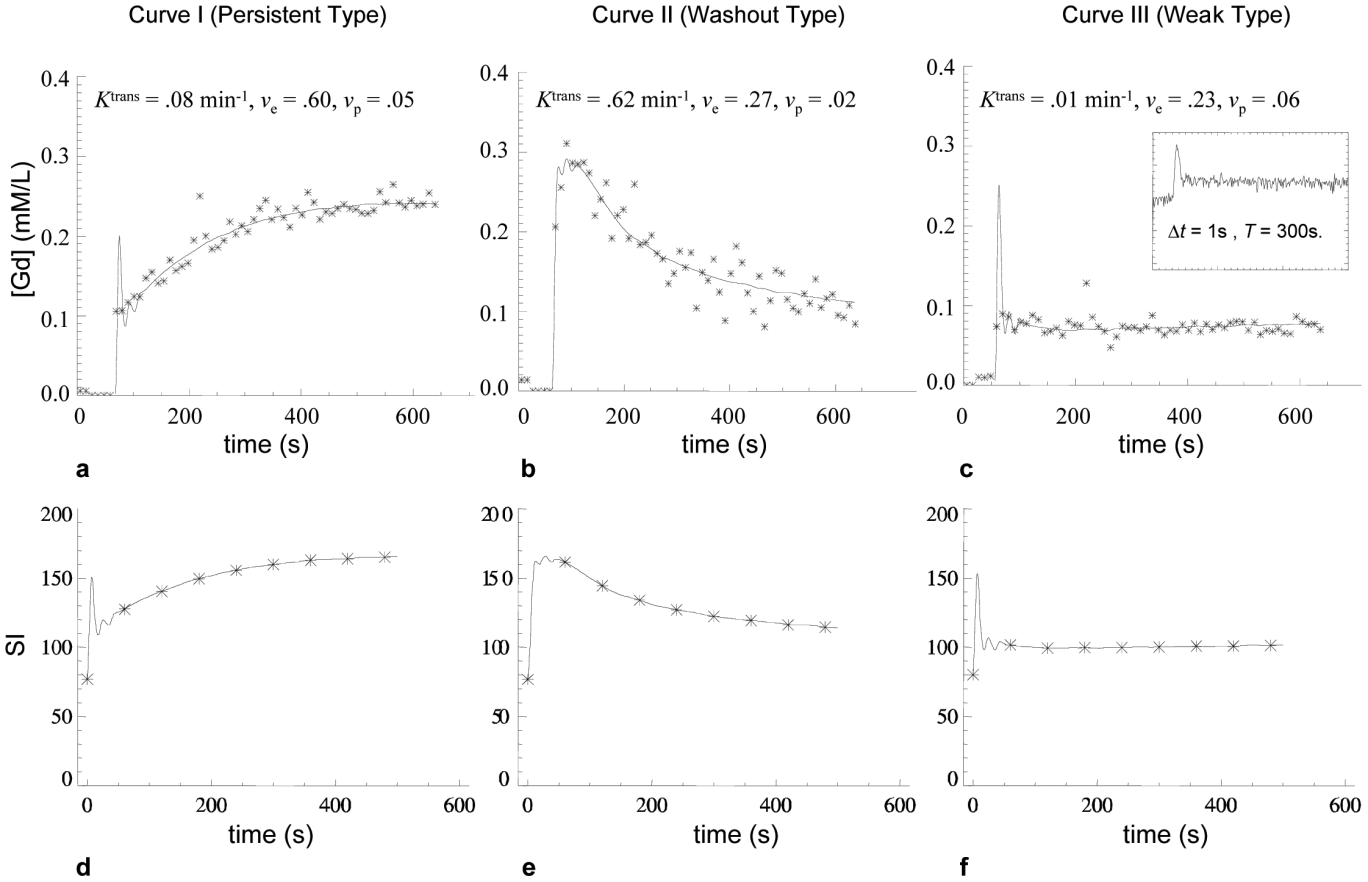
Shows examples of the three typical kinetic enhancement curves used for modelling. curve I (persistent type), curve II (washout type), and curve III (weak enhancement). (**a–c**) demonstrate the [Gd] curves fitted with the modified Tofts model using the ICR-DICE method. The fitted kinetic parameter values are given above each curve. The small panel at the up-right corner of (**c**) shows the SI curves from the corresponding region in the high-temporal resolution (Δ*t* = 1 s) pre-bolus data set of the ICR-DICE acquisition. (**d–f**) demonstrate the SI curves converted from the fitted [Gd] curves. A 1-minute temporal resolution was used to extract data from the theoretical SI curves (showed as asterisk points), which are used as the ‘true’ SI enhancing curves in the following Monte Carlo simulations.

Three patterns of signal intensity time course curves (SI-TC) were chosen for Monte-Carlo analysis [19]: 1) persistent enhancement, 2) contrast washout, and 3) weak contrast enhancement. These represent the three principle variants that have biological value in published studies of SQ parameters. Examples of each type were identified in high-spatial resolution DCE-MRI data of a vestibular schwannoma (VS) in one patient (vide infra). These exemplar signal-time course curves were used to generate a tissue model by:

1. Converting SI-TC to contrast concentration time course curves (CC-TC).
2. Fitting with the modified Tofts model [43], [44].
3. Generating synthetic SI-time curves from the derived fitting parameters using measured baseline SI and pre-contrast T1 relaxation time (*T*_10_), and a literature value of the longitudinal relaxivity of the contrast medium (4.39 mM^−1^ sec^−1^) [45] (Figs 1a–c).
4. Sampling the synthetic time course data with a 1-minute temporal resolution for use as “ground truth” signal intensity-time course curves (SI-TC) in subsequent Monte Carlo analyses (Figs 1 d–f).

#### Monte Carlo simulation for error analysis

MSE_1min_, MSE_rel,1min_, ∑MSE, ∑MSE_rel_ and R_mse1/mse2_, were calculated for each of the reconstructed SI-time curves to produce ‘true’ values (where mse1 = MSE_1min_, mse2 = MSE_5min_ and the sum of MSE and MSE_rel_ were performed over a 5 minute duration). Rician white noise with noise levels ( =  standard deviation/mean baseline signal) of 5%, 10%, and 15% was subsequently added to the simulated SI-TC and SQ parameters calculated to produce ‘measured’ values. A total of 10^6^ repetitions were performed for each given condition (i.e., a specific SI-TC type), and a given noise level. Percent deviations (PD) of the ‘measured’ from ‘true’ values were calculated. Frequency distributions of the percentage deviations were displayed as histograms and distribution characteristics including measures of distribution position (the mean and the median), dispersion (the range, percentiles, and SD), skewness, and kurtosis were calculated. Rank statistics (the range, the 5^th^ and 95^th^ percentiles, and the median) of the PD distributions were displayed using box-and-whisker plots.

#### Monte Carlo simulation for optimal number of pre-contrast time points

The use of a mean value of multiple pre-contrast time points as SI_pre_ for calculation of SQ parameters will reduce the effects of noise and improve reliability [46]. To determine the optimal number of the pre-contrast time points, the above Monte Carlo simulation was repeated but with varying number of pre-contrast time points based on 15% noise level (a typical noise level in our data). 10^4^ repetitions were performed for each given condition (i.e., a specific SI-TC type), and a given number of pre-contrast time points.

### Clinical Studies

#### Ethical statement

The clinical study received approval from the NHS Health Research Authority National Research Ethics Service, North West Committee, Greater Manchester Central, Rec Reference 13/NW/0131 and all patients gave written informed consent for inclusion in the study. All imaging data is archived within the CRUK-EPSRC cancer imaging centre in Cambridge and Manchester archival database and is available to external investigators in anonymized form.

#### Clinical data acquisition

DCE-MRI data were collected in five patients with type 2 neurofibromatosis (NF2), with a total of 12 tumors (7 vestibular schwannoma (VS) and 5 meningiomas). Patients were treated with the anti-vascular endothelial growth factor antibody bevacizumab (5 mg/kg fortnightly, Avastin, Hoffman La-Roche, CH) and were imaged on 3 occasions: pre-treatment (day 0), 3 days (day 3), and 3 months (day 90) following treatment.

DCE-MRI data were collected as described previously [38] using a dual injection technique with an initial high-temporal resolution (1 s), low-spatial resolution acquisition for measurement of the arterial input function followed by a low-temporal, high-spatial resolution (voxel size  = 1×1×2 mm) acquisition for measurement of the tissue residue function. Contrast agent (CA; gadoterate meglumine; Dotarem, Geurbet S.A.) was administered by power injector as an intravenous bolus at a rate of 3 ml/s, followed by a chaser of 20 mls of 0.9% saline administered at the same rate. A low dose of CA (0.017 mmol/kg) was used for the first, high temporal resolution acquisition. For the second, high spatial resolution acquisition a standard dose (0.1 mmol/kg) was administered synchronized with 7^th^ frame of the dynamic acquision yielding six pre-contrast time points in each SI(t) curve.

### Validation of Monte Carlo Error Predictions

Single pixel DCE-MRI data from pre-treatment scans of all tumors (7 VS and 5 meningiomas) was pooled in order to test the predictions of the Monte-Carlo modeling process. Surrogate “true values” of SQ parameters were developed using the assumption that: *where CC-TC data shows good agreement with the modified Toft's model, the resulting fitted function represents the true underlying CC-TC free of noise effects*. For this purpose the residual autocorrelation function and fraction of modeling information (FMI) [47] is a proper quality control for defining the denoised SI curves.

We therefore use the following approach:

- Data for all tumor voxels (n = 117,527) was fitted using a modified Toft's model and estimated values of *K^trans^*,*v_e_* and *v_p_* were derived.
- Voxels where the total error is dominated by modeling error effects were excluded by examining the FMI (Voxels with FMI≤0.995 were excluded).
- The majority of tumor voxels under this study had a noise level of 0.08 to 0.17, corresponding to the simulated data of 0.10–0.15 noise levels. Voxels with noise level outside the range of 0.08–0.17 were therefore excluded.
- Voxels with SI(*t*) curves resembling the typical persistent enhancement (0.03<*K* ^trans^<0.09 min^−1^, 0.55<*v*_e_<0.90, and 0.001<*v*_p_<0.07) and typical washout patterns (0.3<*K* ^trans^<1.0 min^−1^, 0.20<*v*_e_<0.45, and *v*_p_<0.15) were identified. The Ranges for values of Ktrans, ve and vp for the persistent and washout curves were setup with the consideration of obtaining enough in vivo curves for the validation while keeping the typical persistent or washout type.
- Theoretical SI-TC were generated for the selected voxels using the measured PK parameters and *T*_10_ values of the corresponding voxels.
- SQ parameters were calculated from the theoretical SI-TC to serve as “true values”.

**Figure 2:**
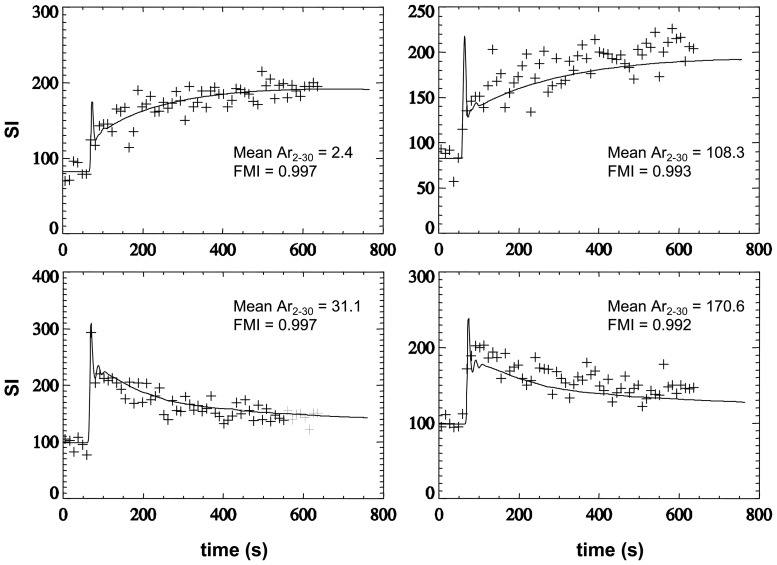
Shows fitting results for Experimental and modelled signal enhancement data SI(*t*). Experimental data are shown with symbols of “+”. Modeled data are shown as a solid line. The good fits (left column) are characterized by a lower (near zero) ‘Mean Ar_2–30_’ and a higher fraction of modeling information (FMI) [47], where Ar is the autocorrelation of the residual, and the ‘Mean Ar_2–30_’ was the mean Ar over the time lags 2 to 30. The poor fits with considerable modeling error (right column) are characterized by a higher ‘Mean Ar_2–30_’ and a lower FMI.

Figure 2 shows examples of curves used in the PD distribution analysis (left column) and of those excluded (right column) based on the temporal autocorrelation analysis.

These voxel selection criteria, outlined above, were used to select a subgroup of voxels whose enhancement curves corresponded very closely to the three principal subtypes identified in the Monte Carlo simulation. In addition, although most of the voxels in the tumors could be fitted with a scaled fitting error (SFE) around 15% or lower, only around one third of the typical persistent or typical washout curves could be fitted with a fraction of modeling information (FMI) equal to, or higher than, 0.997 and these voxels were used in the validation. This resulted in the exclusion of the majority of voxels that were represented by mixed curve types leaving 8291 voxels with persistent and 448 with washout type curves. This selection process is necessitated to support direct comparison with the three main curve types selected for the Monte Carlo simulation. We therefore divided the 10^6^ Monte Carlo simulated persistent type curves into 100 groups (each has 10,000 samples), and the 10^6^ simulated washout type curves into 2000 groups (each has 500 samples). PD distributions were calculated for each of the subgroups. A range of values for each of the descriptive statistics of the PD distribution were produced and used for comparison to the in vivo data.

#### Comparing therapy-induced changes in PK and SQ parameters

DCE-MRI datasets from pre-treatment, 3 days and 3 months post treatment were analysed for each patient. Tumors were automatically segmented using high-spatial resolution 3D Bayesian probability maps [48]. 3D Parametric maps of the five SQ parameters and of *K* ^trans^, *v*_p_, *v*_e_, and rate constant *k*_ep_ (≡*K* ^trans^/*v*_e_), calculated using the modified Tofts model [43], [44], were generated from each data set. Calculations of SQ parameters used a pre-contrast measurement consisting of an average of 5 pre-contrast baseline measurements.

Pre- and post-treatment differences in SQ parameters were tested using two samples Wilcoxon rank-sum test for VS and meningiomas respectively. Spearman's rank order correlation was used to analyze the relationship between *k^t^* ^rans^ and each of four SQ parameters (MSE, MSE_rel_, ∑MSE, ∑MSE_rel_) across two tumor groups, i.e. VS and meningioma for each visit.

Since a close correlation between R_mse1/mse2_ and *k*ep has been reported by previous workers [12], [31], we used Spearman's rank order correlation to analyze the relationship between R_se1/se2_ and *k*_ep_ across the two tumor groups for each visit and also performed a pixel-by-pixel comparison of the pre- and post-treatment values of R_mse1/mse2_ and *k*ep using scatter plots and linear regression analyses.

## Results

### Monte Carlo Simulation

#### 1. What is the predicted effect of variations in signal intensity curve shape and signal to noise ratio on errors in the estimates of SQ parameters?

**Figure 3:**
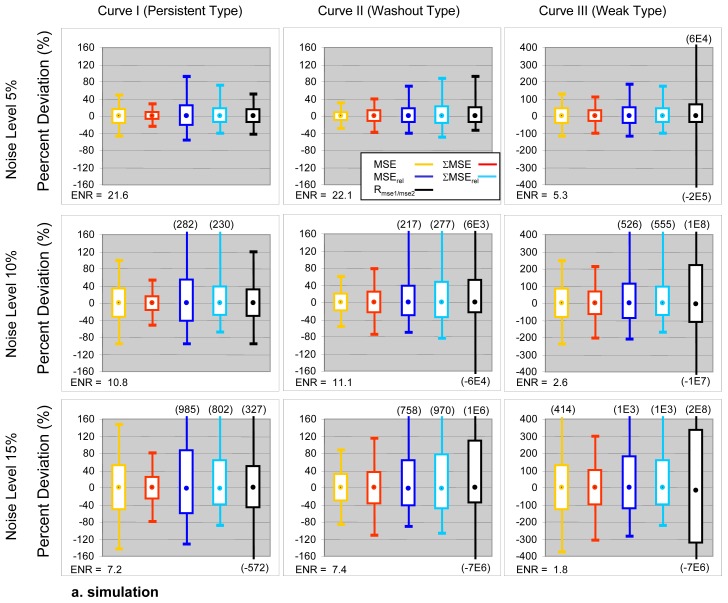
Box-and-whisker plots showing the PD distributions of the five empirical parameters. The range between 5^th^–95^th^ percentiles is shown by the box. Extreme values are shown by whiskers. Median is shown by a dot within the box. The PD distributions calculated from 1000000 Monte Carlo repetitions. ENR for each simulated condition are annotated. Formula for calculation of ENR can be found in Table 1.

Measures for distribution characteristics of PD values from the 10^6^ Monte Carlo repetitions are shown in Table 1 and Figure 3. Table 2 compares the descriptive statistics of PD distributions from Monte Carlo simulations and in vivo data. The in vivo values for all descriptive statistics, in general, lie within the range (minimum and maximum) given by the Monte Carlo simulated PD distributions.

**Table 1:** Descriptive statistics for PD distributions calculated from 10^6^ Monte Carlo repetitions for each of the five SQ parameters under varying noise and pharmacokinetic conditions.

	PD	Mean			SD			Skewness			Kurtosis		
Curve type	noise(%)	5	10	15	5	10	15	5	10	15	5	10	15
Type I: Persistent	MSE	−0.1	−0.3	−0.7	10.7	21.3	31.9	0.0	0.0	0.0	0.0	0.0	0.0
	ΣMSE	−0.1	−0.2	−0.5	5.3	10.6	15.9	0.0	0.0	0.0	0.0	0.0	0.0
	MSE_rel_	0.4	1.8	4.0	14.7	30.0	46.6	0.3	0.5	0.9	0.1	0.7	2.2
	ΣMSE_rel_	0.3	1.3	3.0	10.3	21.0	32.8	0.3	0.6	1.1	0.2	0.8	3.0
	R_mse1/mse2_	0.1	0.3	0.7	9.4	19.2	30.1	0.1	0.1	0.2	0.1	0.3	1.1
Type II: Washout	MSE	−0.1	−0.2	−0.5	6.4	12.8	19.1	0.0	0.0	0.0	0.0	0.0	0.0
	ΣMSE	−0.1	−0.3	−0.6	7.7	15.3	22.8	0.0	0.0	0.0	0.0	0.0	0.0
	MSE_rel_	0.3	1.2	2.8	10.6	21.6	33.7	0.3	0.6	1.0	0.1	0.7	2.4
	ΣMSE_rel_	0.4	1.7	3.9	12.6	25.7	40.2	0.3	0.6	1.1	0.2	0.8	2.9
	R_mse1/mse2_	1.1	5.2	6.8	10.9	69.6	8E3	0.7	−622	−801	1.0	5E5	7E5
Type III: Weak	MSE	−0.1	−0.4	−1.0	26.7	53.4	79.8	0.0	0.0	0.0	0.0	0.0	0.0
	ΣMSE	−0.1	−0.3	−1.0	21.1	42.2	63.1	0.0	0.0	0.0	0.0	0.0	0.0
	MSE_rel_	1.0	4.0	9.0	30.6	62.3	96.6	0.2	0.5	0.9	0.1	0.6	1.9
	ΣMSE_rel_	1.1	4.2	9.4	26.1	53.4	83.5	0.3	0.6	1.1	0.2	0.8	2.6
	R_mse1/mse2_	6.0	134	192	253	1E5	2E5	−465	945	954	3E5	9E5	9E5

**Table 2:** Comparison of the SQ parameter PD distributions calculated from Monte Carlo simulations and the in vivo data.

	PD Distribution in		Min	5^th^ Perc.	Median	95^th^ Perc.	Max
Persistent	MSE	Simul.^a^	[−145, −104]	[−55, −52]	[−2, 1]	[50], [53]	[105, 147]
		In vivo	−126	−54	−1	55	155
	ΣMSE	Simul.^a^	[−79, −54]	[−28, −26]	[−1, 0]	[25], [26]	[51, 80
		In vivo	−48	−20	3	27	71
	MSE_rel_	Simul.^a^	[−133, −103]	[−62, −59]	[−3, −1]	[84, 92]	[257, 988]
		In vivo	−121	−59	−1	81	529
	ΣMSE_rel_	Simul.^a^	[−88, −68]	[−43, −40]	[−3, −1]	[60], [65]	[192, 802]
		In vivo	−58	−32	3	55	429
	R_mse1/mse2_	Simul.^a^	[−572, −105]	[−49, −45]	[−1, 1]	[49], [52]	[139, 327]
		In vivo	−136	−50	−3	50	209
Washout	MSE	Simul.^b^	[−87, −42]	[−39, −25]	[−4, 3]	[25], [37]	[39, 88]
		In vivo	−67	−32	5	46	82
	ΣMSE	Simul.^b^	[−113, −51]	[−47, −30]	[−5, 4]	[29], [45]	[46, 115]
		In vivo	−59	−35	2	47	118
	MSE_rel_	Simul.^b^	[−92, −53]	[−50, −35]	[−7, 4]	[49, 86]	[90, 758]
		In vivo	−72	−38	7	71	199
	ΣMSE_rel_	Simul.^b^	[−108, −64]	[−60, −42]	[−9, 5]	[59, 102]	[111, 970]
		In vivo	−65	−43	3	86	283
	R_mse1/mse2_	Simul.^b^	[−7E6, −44]	[−42, −31]	[−6, 5]	[70, 186]	[210, 1E6]
		In vivo	−1577	−45	11	148	1862

a Simul.(Simulated) showed by the range of minimum and maximum from 100 Monte Carlo repetitions.

b showed by the range of minimum and maximum from 2000 Monte Carlo repetitions.

#### 2. Do observations from the clinical DCE-MRI data support Monte-Carlo predictions?

Simulation and in vivo data demonstrate close agreement and show the following:

1. Parameters “normalized” using pre-contrast signal intensities show poorer precision than non-normalized metrics. SD values of PD distributions of normalized metrics were 37%−94% greater than their non-normalized counterparts at noise level of 5% and 46%−106% greater at noise level of 15%. In vivo data showed that the 90% confidence ranges (range 5^th^ to 95^th^ percentiles) of the PD for normalized metrics were 22%−85% greater than their non-normalized counterparts and 82%−309% greater in the extreme range (range between the minimum and maximum values) (Table 2) (23).
2. ∑MSE and ∑MSE_rel_ show greater precision for persistent and lower precision for washout type curves when compared to MSE_1min_ and MSE_1min, rel_. SD values of PD distributions of ∑MSE and ∑MSE_rel_ were 50% and 30% less than for MSE_1min_ and MSE_1min, rel_ for persistent but 19%−20% greater than their non-sum counterparts for washout type curves (Table 1). In vivo data confirmed these predictions showing that the SD of PD for ∑MSE and ∑MSE_rel_ were 57% and 38% less than MSE_1min_ and MSE_1min, rel_ for persistent but 5% and 18% greater for washout type curves in the 90% confidence ranges of the PD (Table 2).
3. Both simulation and in vivo data showed that: (1) PDs in MSE and ∑MSE are normally distributed (K-S test, p>0.05); (2) PD distributions normalized MSE and ∑MSE show a right skew although the K-S test was not significant, 3) the PD distribution in R_mse1/mse2_ is not normal with fat tails in the PD distributions [49] (K-S test (p<0.001).

#### 3. Does the behavior of the signal enhancement ratio (R_mse1/mse2_) provide a satisfactory surrogate metric for formal PK based analyses?

**Figure 4:**
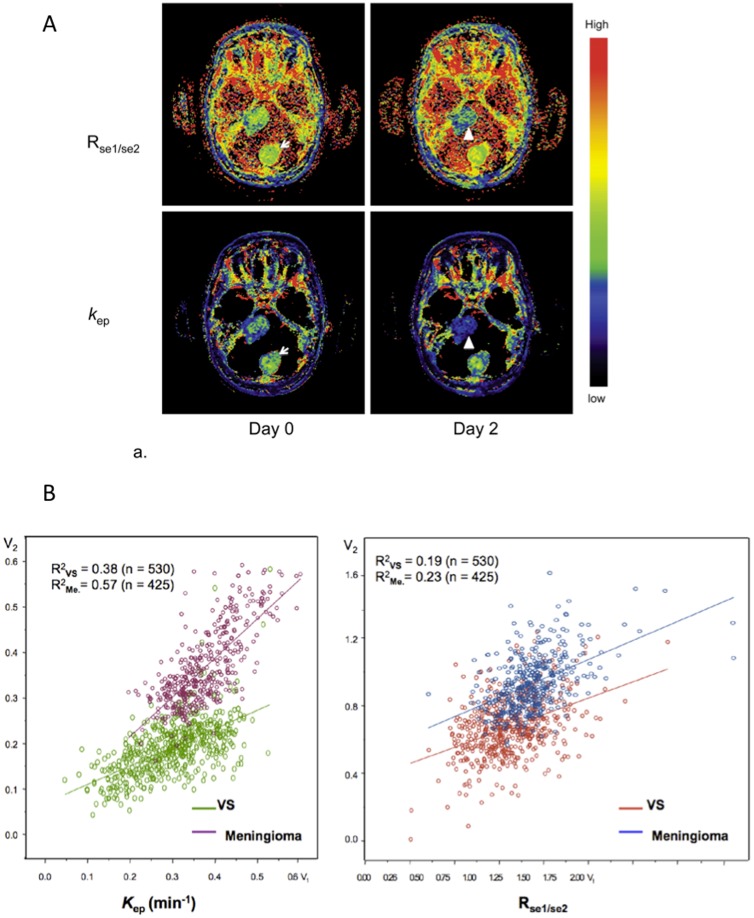
Demonstrating direct comparisons between R_mse1/mse2_ and *k*_ep_ in response to treatment in a single patient. (**a**) Longitudinal spatially co-registered maps of R_mse1/mse2_ (top) and *k*_ep_ (bottom) before (left) and 3 days after bevacizumab treatment (right) from a central slice of the tumors. (**b**) Pixel-by-pixel scatter plots of the parametric values (3 days post-treatment against the pre-treatment) produced from the right vestibular schwannoma and the meningioma on the same slice as in (a). The values of R_mse1/mse2_ were calculated with an average of the five pre-contrast baseline measurements as SI_pre_, MSE_1_ = MSE_1min_ and MSE_2_ = MSE_5min_. Mean *k*_ep_ of VS was 0.30±0.10 on day 0 and 0.19±0.08 on day 3. Mean *k*_ep_ of meningioma was 0.38±0.09 on day 0 and 0.36±0.09 on day 3. Mean R_mse1/mse2_ of VS was 0.82±0.22 on day 0 and 0.68±0.19 on day 3. Mean R_mse1/mse2_ of meningioma was 0.94±.27 on day 0 and 0.94±0.18 on day 3.

Previous studies have recommended the use of R_mse1/mse2_ because it shows correlation with *k*_ep_ [12], [31]. In the current study R_mse1/mse2_ was significantly correlated with *k*_ep_ on day 0 (p = 0.026) and day 90 (p = 0.004), although this relationship was not observed on day 3 (p>0.05). Figure 4a shows longitudinal coregistered R_mse1/mse2_ and *k*_ep_ maps on day 0 and day 3. Scatter plots of *k*_ep_ pixel values from day 0 and day 3 show correlation (Fig. 4b: *R* ^2^_VS_ = 0.38; *R* ^2^_meningioma_ = 0.57) with a clear change in the slope of the regression line reflecting treatment induced change in VS (*k*_ep, post_ = 0.48, *k*_ep, pre_+0.05) but no change in meningioma (*k*_ep, post_ = 0.94, *k*_ep, pre_+0.00). Despite the expected correlations between the two parameters the scatterplot for R_mse1/mse2_ shows weak correlations (Fig. 4: *R* ^2^_VS_ = 0.19; *R* ^2^_meningioma_ = 0.23) with reduced separation between VS and meningioma.

In modeling studies R_mse1/mse2_ showed the greatest variability in response to noise and PK conditions. R_mse1/mse2_ was much more robust for persistent than for washout type curves. Simulations showed that SD values of the PD distributions for washout were 16%, 263% and 2E4% greater than those of persistent type curves for noise levels of 5%, 10%, and 15% respectively (Table 1, Fig 3). In vivo data confirmed this behavior showing SD values for washout 93% greater than for persistent type curves in the 90% confidence ranges of the PD, and 897% greater in the extreme range (Table 2).

Comparison of values in mse1, mse2, and R_mse1/mse2_ maps demonstrated that 3−4% of voxels have spurious values resulting from noise-induced near-zero denominator (se2) values in the ration calculation. To remove these outliers, a thresholds of 0<R_mse1/mse2_<1.45 was used to exclude voxels outside the 95^th^ percentile. The denoised R_mse1/mse2_ (R_mse1/mse2 (DN)_) demonstrates treatment–induced changes in VS similar to those seen with other SQ metrics. Post-treatment R_mse1/mse2 (DN)_ of VS 3 and 90 days after therapy were significantly smaller than pre-treatment (p = 0.017; p = 0.026), whilst post-treatment R_mse1/mse2 (DN)_ of meningiomas at day 3 showed no change (p>0.05). Post treatment R_mse1/mse2(DN)_ at day 90 of meningiomas were significant smaller than pre-treatment (P = 0.008). Spearman's rank correlations showed R_mse1/mse2 (DN)_ showed close positive correlation with *k*_ep_ for all three visits.

#### 4. What is the optimal number of samples to define pre-enhancement signal intensity on estimates of SQ parameters?

The Monte-Carlo simulation showed that, with a 15% noise level (a typical noise level in our data), two pre-contrast time frames improved the precision of SQ parameter estimation with a resulting reduction in the standard deviation of PDs of 13%, 24%, 25%, and 31% for MSE, MSE_rel_, ∑MSE, and ∑MSE_rel_ respectively for persistent type curves; 13%, 26%, 25%, and 31% for washout type curves. The improvement started from 2 and came to a plateau at time frame 6. The PD distributions of normalized metrics were more affected with reduction of positive skewness and kurtosis. The precision of R_mse1/mse2_ was least affected by increasing number of pre-contrast time points for persistent type curves, but most affected for washout type curves.

#### 5. How do SQ and PK derived metrics compare in the detection of anti-angiogenic therapy induced changes in a single centre setting?

**Figure 5:**
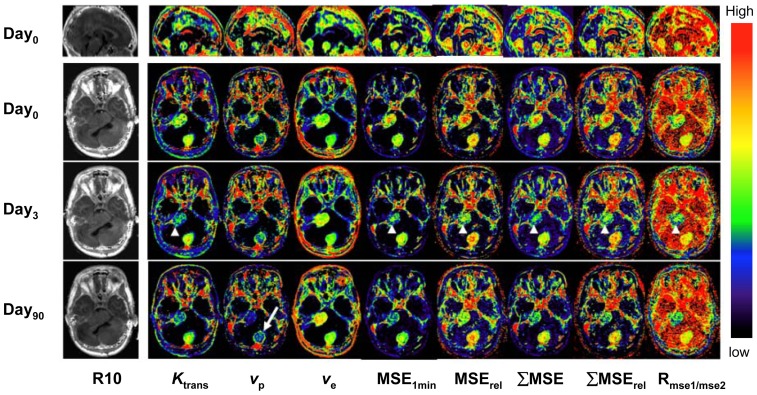
Axial view of central slices of 3D longitudinally co-registered kinetic and semi-quantitative parametric maps obtained in 26-year-old woman, who has had a progressive VS (arrow heads) and an occipital located meningioma (arrow) undergoing treatment with bevacizumab. Note that a ring-like structure on the margin of the meningioma can only be seen on the *v*_p_ maps (arrow), but not on any of semi-quantitative maps.

**Figure 6:**
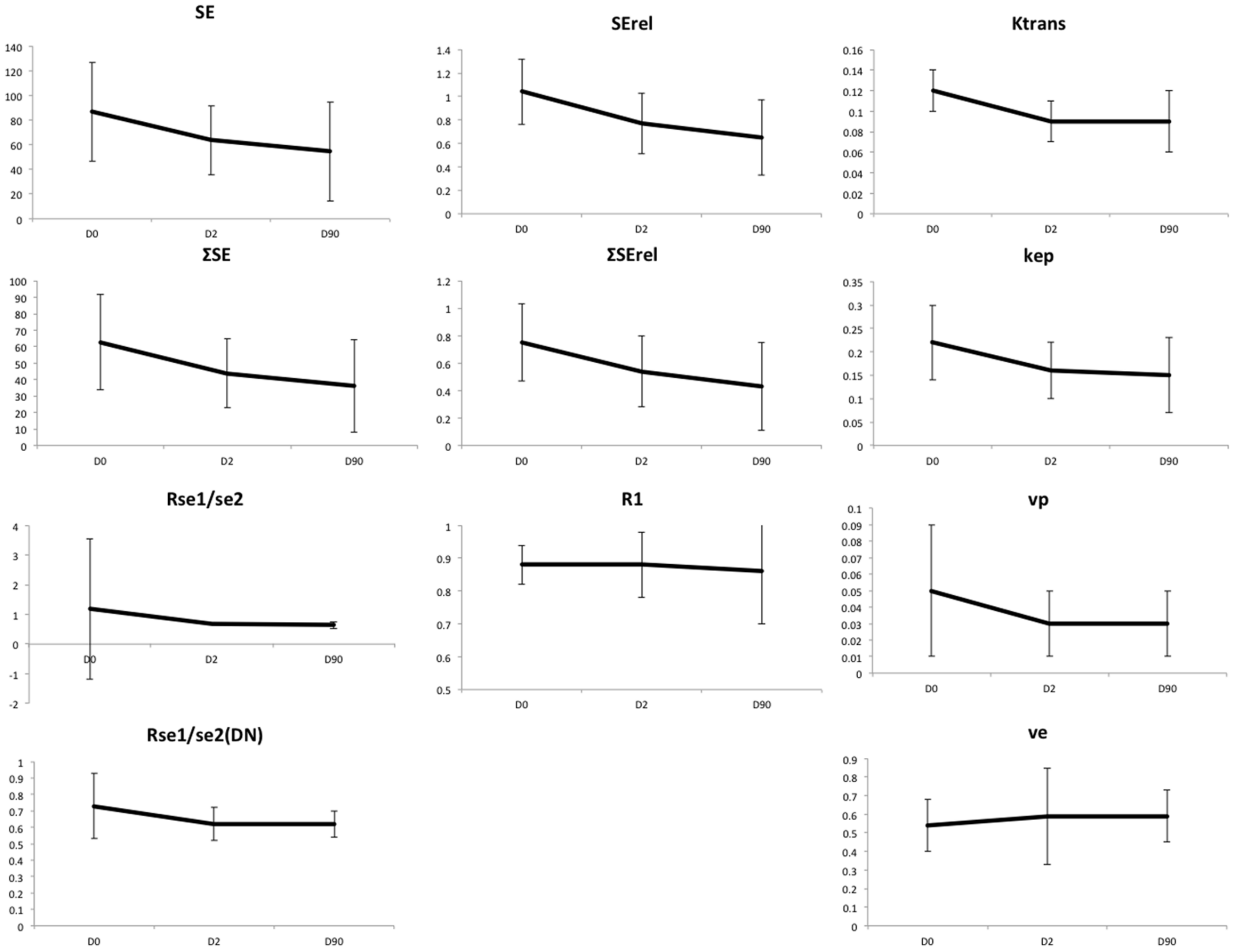
Plots of the changes in each of the SQ and PK parameters for VS during treatment. Error bars represent 95% confidence limits. Significant change compared to pre-treatment (paired comparisons) is indicated by stars, * = p<0.05, ** = p<0.01.

Parametric maps of both PK and SQ parameters were of high quality (Fig 5). Table 3, and Figure 6 show mean values of PK and SQ parameters in pre and post-treatment tumors. There were no significant changes of longitudinal relaxation rates *R*_1_ (≡1/*T*_1_) in VS or meningiomas after treatment. In VS; *K* ^trans^, v_p_, MSE, MSE_rel_, ΣMSE and ΣMSE_rel_ and R_mse1/mse2(DN)_ measured on day 3 and day 90 were significantly lower than at day 0. From the SQ parameters, only R_mse1/mse2_ showed no significant changes in response to therapy. From the PK parameters v_e_ showed no significant treatment related change. In meningiomas; R_mse1/mse2(DN)_ measured 90 days after treatment was significantly lower than at day 0. Other than this there was no significant post-therapy change of PK (i.e. *K* ^trans^, v_p_. v_e_) or SQ (i.e. MSE, MSE_rel_, ΣMSE, ΣMSE_rel_, R_mse1/mse2_) parameters in meningiomas.

**Table 3:** Comparison of mean values of DCE parameters in pre- and post-treatment VS and Meningiomas.

		^b^Compare Drug-Induced Changes of Mean in Two Tumors Wilcoxon Rank-sum Test		
Tumor type	^a^DCE- MRI Para-meters.	Day 0	Day 3	Day 90
VS	MSE	86.7±20.2	**63.6±14.0** *	**54.4±19.7** *
	MSE_rel_	1.04±0.19	**0.77±0.17** *	**0.65±0.23** **
	ΣMSE	62.6±14.5	**43.7±10.4** *	**36.0±14.0** *
	ΣMSE_rel_	0.75±0.14	**0.54±0.13** *	**0.43±0.16** **
	R_mse1/mse2_	1.18±1.18	0.68±0.01	0.64±0.06
	R_mse1/mse2(DN)_	0.73±0.10	**0.62±0.05** *	**0.62±0.04** *
	*R*_1_	0.88±0.03	0.88±0.05	0.86±0.08
	*K* ^trans^	0.12±0.01	**0.09±0.01** **	**0.09±0.03** *
	*v*_p_	0.05±0.02	**0.03±0.01** *	**0.03±0.01** *
	*v*_e_	0.54±0.07	0.59±0.13	0.59±0.07
	*k*_ep_	0.22±0.04	**0.16±0.03** **	**0.15±0.04** **
Meningi-omas	R_mse1/mse2_	1.00±0.08	4.91±38.2	**0.85±0.04** **
	R_mse1/mse2(DN)_	0.98±0.06	0.93±0.09	**0.84±0.03** **

a MSE, ΣMSE are in arbitrary unit, MSE_rel_, ΣMSE_rel_ and R_mse1/mse2_, *k*_ep_ are ratios. *K* ^trans^ is in minute^−1^.

b Comparison of differences in DCE-MRI parameters between pre- and post-treatment in each group of tumors, i.e. VS (N = 7) and meningiomas (N = 5) for each pair of visits (Day 0 and day 3, day 0 and day 90).

*p<0.05,

**p<0.01.

Table 4 lists correlation coefficients and its significance level where the relationships between K^trans^ and SQ parameters, i.e. MSE, MSE_rel_, ΣMSE and ΣMSE_rel_, were tested using Spearman's rank order test. Mean values of MSE, MSE_rel_, ΣMSE and ΣMSE_rel_ in VS and Meningiomas were all significantly correlated to mean values of *K* ^trans^ on day 0, day 3 and day 90 (*p* = 0.002−0.042) with the exception of ΣMSE_rel_ measured on day 90, which showed only a trend of relationship towards *K* ^trans^ (*p* = 0.076).

**Table 4:** Spearman's rank order correlation between K^trans^ and SQ parameters, i.e. MSE, MSE_rel_, ΣMSE and ΣMSE_rel_.

	MSE		MSE_rel_		∑MSE		∑MSE_rel_	
	Est.*	p-val	Est.	p-val	Est.	p-val	Est.	p-val
Pre-treatment	0.776	0.010	0.734	0.015	0.699	0.020	0.650	0.031
Day 3	0.839	0.015	0.885	0.003	0.692	0.022	0.727	0.016
Day 90	0.937	0.002	0.818	0.007	0.609	0.044	0.538	0.076

*Est.: Estimate.

## Discussion

DCE-MRI has become one of the most widely used techniques for the characterization of tissue microvasculature with particularly wide uptake in oncology [3], [4], [50], [51]. Despite this there remain significant problems with application and dissemination of the technique, largely resulting from variability between imaging platforms. In clinical trials this has led to a dominance of PK based analysis techniques since it is reasoned, probably correctly, that the use of calculated contrast concentration in place of SI data reduces a major aspect of variation [3], [4]. However the use of PK metrics introduces additional problems including the need to define an accurate arterial input function (causing competing demands for temporal and spatial resolution), difficulties with measurement of pre- and post- treatment T1 values required for calculation of contrast concentration, the choice of appropriate pharmacokinetic models and the need for curve fitting analyses. The PK approach is therefore complex and, until recently, has been largely limited to individual centre studies [52]. As a consequence there is wide variation in acquisition and analysis approaches [51]. It is surprising that, 20 years after the introduction of DCE-MRI there is no standardization of acquisition sequences or analytical software for clinical or research use despite extensive investment in time and resources by academic groups, government and the pharmaceutical industry [1], [3], [4], [37], [53], [54], [55].

Previous investigators showed the diagnostic importance of the shape of time–signal intensity curves [56] in differentiating benign and malignant enhancing lesions in breast [19], [57], [58], hepatic Lesion [59], and lesions of bone [60], brain [27], colorectal [61] and prostate [62]. Some studies analyzed correlation of CA enhancement patterns on MR images with histopathological findings and tumor angiogenesis [63], [64], and with PET imaging[65]. This study analyzed error propagation of SQ parameters in three types of SI-time courses, selected on the basis of these published findings, to investigate these curve shapes' preference for robust calculation of SQ parameters.

SQ parameters “normalized” using pre-contrast signal intensities are thought to be necessary to reduce the dependency on both biological and imaging variables such as coil filling factor, transmitter and receiver gain etc., which vary from scan to scan and/or patient to patient [18]. As expected, the normalized metrics (MSE_rel_ and ∑MSE_rel_) were found to be more susceptible to noise than their non-normalized counterparts (MSE and ∑MSE) and we have confirmed suggestions by previous workers that the use of multiple pre-contrast time points to obtain a mean value of SI_pre_, significantly improves estimation errors [46]. We have also shown that this benefit is significant when only 2 pre-contrast data points are collected and plateaus at 6. In the therapy setting ∑MSE, MSE, ∑MSE_rel_, and MSE_rel_, correlated with and showed similar behavior to *K* ^trans^, [66], [67]. The predicted and observed error distributions in SQ parameters described here could be considered a disadvantage to their use in clinical trials. However, similar Monte-Carlo modeling analyses of PK analytical approaches have demonstrated not only noise related errors, equal to or greater than those described here, but also systematic bias resulting in very poor precision in K^trans^ at low values, particularly the presence of low SNR [29].

The signal enhancement ratio (R_mse1/mse2_) is little affected by variation in tissue *T*_10_ values and has been shown to correlate closely with the redistribution rate constant (*k*_ep_), a commonly analyzed PK parameter [31]. This makes R_mse1/mse2_ a potentially attractive and very widely used metric, particularly in breast cancer studies [9], [10], [11], [12], [13], [32], [33], [34], [35], [36]. However, in the majority of these studies R_mse1/mse2_ was measured on an averaged SI curve from a region of interest producing high SNR [12]. We have shown that single pixel mapping is associated with poor SNR and considerable heterogeneity in data quality. We demonstrated that R_mse1/mse22_ has poor tolerance of low SNR, which is particularly severe when it is calculated from washout type curves. The outliers identified from both simulation and in vivo R_mse1/mse2_ lead to high kurtosis and skewness in the PD distribution function. It is clear that these outliers should be carefully treated prior to statistical analysis. It is encouraging that, as shown in this study, the method using 95^th^ percentile as a threshold for the removal of outliers has demonstrated efficacy in restoring the power of R_mse1/mse2_, in detecting the therapy-induced changes and resulted in the demonstration of therapy induced changes in meningiomas which were not detected by other SQ or PK parameters. Use of an R_mse1/mse2_ histogram based volume analysis is an alternative approach that is also less affected by outliers [11].

One of the major stated benefits of the PK analytical approach is the physiological specificity of the metrics. When DCE-MRI studies of antiangiogenic therapies became common, K^trans^ was widely used as an indicator of changes in endothelial permeability. In fact it represents a compound metric affected by both blood flow and the product of the surface area and permeability of the capillary endothelial membrane. Nonetheless, the use of K^trans^ as a biomarker effectively removes confounding effects due to variations in *v_p_* and *v_e_*. The statistical power of any given metric to detect significant change is dependent both on the change in mean/median value and, more importantly on the shape and width of the data distribution. In this study, the group coefficients of variation for SQ metrics in VS ranged from 18–24%. However, the CoV for K^trans^ was considerably less both at day 0 and at 3 days of treatment (COV 8.3 and 11% respectively). This was associated with very high COV for *v_p_* (40 and 33% respectively). This narrow distribution resulted in improved statistical power for K^trans^ compared to SQ metrics such that a 25% reduction in K^trans^ at day 90 produced similar significance values to 37%, 37%, 42% and 43% decreases in MSE, MSE_rel_, ∑MSE and ∑MSE_rel_ respectively. The reasons for this difference in COV is not clear, however it seems likely that the systematic removal of the contributions of intravascular contrast (v_p_) removed a significant source of variation from the estimated mean tumor values. Interestingly, the denoised signal enhancement ratio (R_mse1/mse2(DN)_) also demonstrated low COV (13.7%, 8.0% and 6.4% at days 0,3 and 90) and showed treatment induced changes of similar significance to K^trans^ despite a reduction of only 15.1% in mean values. In meningiomas pre-treatment K^trans^ had the largest COV of any metric other than v_p_ (40% and 60% respectively). R_mse1/mse2 (DN)_ showed the lowest COV (6.1%, 9.6%, and 3.6% at days 0, 3 and 90) and was the only metric to demonstrate significant therapy induced changes with a reduction in mean values of 14% compared for example to non-significant reduction in mean *K_ep_* of 13%. The R_mse1/mse2(DN)_ parameter is also designed to remove confounding numerical effects from estimates of the mean value. These results demonstrate the importance of variation in the mean estimated values of each parameter across the tumor population and favour the use of parameters where variation is minimized by choice of the appropriate metric, whether SQ or PK. Unfortunately the difference in behavior between meningiomas and VS shows that the potential variation in individual metrics may be tumor specific and cannot be predicted during trial design. These observations also support the suggestions of previous workers that analysis of DCE-MRI data should ideally be performed on a pixel by pixel basis [2], [3] and that the effects of spatial heterogeneity should be specifically included into the analytical approach [68].

In conclusion, this study begins to challenge the commonly expressed criticisms of SQ metrics for pixel-by-pixel parametric mapping and clinical trials. The SQ parameters in this study show relatively high tolerance to poor SNR compared to previous studies of PK metrics and demonstrated equivalent therapy induced changes. This paper is deliberately controversial and the majority of reviews and consensus workshop reports on DCE-MRI favor PK over SQ metrics for the reasons discussed above. However we believe that the results presented here demonstrates some clear advantages of SQ metrics and supports a re-evaluation of the utility of these metrics, which must include comparative studies of baseline reproducibility and multi-vendor/multicenter implementations.
